# Role of arbuscular mycorrhizal fungi in drought-resilient soybeans (*Glycine max* L.): unraveling the morphological, physio-biochemical traits, and expression of polyamine biosynthesis genes

**DOI:** 10.1186/s40529-025-00455-1

**Published:** 2025-03-17

**Authors:** Elham R. S. Soliman, Reda E. Abdelhameed, Rabab A. Metwally

**Affiliations:** 1 Cytogenetics and Molecular Genetics Unit, Botany and Microbiology Department, Faculty of Science, Helwan University, Helwan, 11795 Egypt; 2 Botany and Microbiology Department, Faculty of Science, Zagazig University, Zagazig, 44519 Egypt

**Keywords:** Alkaline phosphatase, Gas exchange parameters, Glomalin, Lipid peroxidation, Osmo-protectants, Soybean, Spermidine synthase, Spermine synthase, Water use efficiency

## Abstract

**Background:**

Drought stress is a catastrophic abiotic stressor that impedes the worldwide output of commodities and the development of plants. The Utilizing biological antioxidant stimulators, Arbuscular mycorrhizal fungi (AMF) are one example increased the plants' ability to withstand the effects of drought. The symbiotic response of soybean (*Glycine max* L.) to AMF inoculation was assessed in the experiment presented herewith at different watering regimes (field capacity of 25, 50, and 90%). The vegetative, physio-biochemical traits, and regulation of genes involved in polyamine synthesis in *G. max* plants were evaluated.

**Results:**

The results obtained suggested that AMF inoculation has an advantage over plants that were non-inoculated in terms of their growth and all assessed criteria, which responded to drought stress by showing slower development. It is evident that the gas exchange parameters of the soybean plant were substantially reduced by 36.79 (photosynthetic rate; *A*), 60.59 (transpiration rate; *E*), and 53.50% (stomatal conductance *gs*), respectively, under severe stress of drought in comparison to control; non-stressed treatment. However, the AMF inoculation resulted in a 40.87, 29.89, and 33.65% increase in *A*, *E*, and *gs* levels, respectively, in extremely drought-stressful circumstances, when in contrast to non-AMF one that was grown under well-watered conditions. The drought level was inversely proportional to mycorrhizal colonization. The total antioxidant capacity, protein, and proline contents were all enhanced by AMF inoculation, while the malondialdehyde and hydrogen peroxide contents were decreased. Polyamine biosynthesis genes expression; Ornithine decarboxylase (ODC2), Spermidine synthase (SPDS) and Spermine synthase (SpS) were upregulated in drought and to even higher level in AMF’s mild drought inoculated plants’ shoots. This implies that AMF plays apart in the enhanced survival of soybean plants stressed by drought and reduced plant membranes damage by limiting the excessive production of oxidative stress generators; ROS.

**Conclusions:**

In summary, the present investigation demonstrates that inoculation of AMF may be a supportable and environmentally advantageous method for improving the physio-biochemical traits, plant growth, and polyamine biosynthesis genes of soybean plants in the incident of limited water availability.

**Supplementary Information:**

The online version contains supplementary material available at 10.1186/s40529-025-00455-1.

## Background

In nature, plants are exposed to a varied range of environmental stimuli that result in detrimental modifications to their patterns of development and growth. Among these constraints, drought is one that induces a sequence of adverse consequences for plants, osmotic imbalance, membrane harm, and complications with metabolism and photosynthetic processes that impede proper growth and metabolism (Hashem et al. 2019**; **Hasanuzzaman et al. 2020**; **Iqbal et al. 2022**; **Li et al. 2022**)**. *Glycine max* L. that is commonly known as soybean is a legume that is cultivated worldwide and is of economic significance. The majority of soybeans are used for their oil and protein constituents **(**Abdalla et al. 2022**)**. This plant's productivity has been diminished by the progressively extended drought periods that have resulted from climate change **(**Cera et al. 2017; Bittencourt et al. 2021**)**. Similar to other abiotic stresses, plants experienced drought periods are induced to produce excessive amounts of (ROS); reactive oxygen species, which results in their overabundance in various subcellular compartments, including plasma membranes, chloroplasts and mitochondria **(**Hasanuzzaman et al. 2020; Li et al. 2022**)**. Their excessive accumulation can result in an oxidative surge, which in turn causes oxidative injury in plants. This oxidative injury has the capacity to denature proteins, cause impairment to nucleic acids, catalyze pigment degradation, increase carbohydrate oxidation, induce programmed cell death, and cause peroxidation of lipids in cellular membranes **(**Hashem et al. 2016; Choudhary et al. 2018; Al-Arjani et al. 2020; Nasrallah et al. 2020**)**.

Plant biological activities, including transpiration rate (*E*) and relative water content (RWC), are diminished by the stress of drought, as Desoky et al. (2023) proved that a considerable decrease in RWC, membrane stability index, and leaf photosynthetic rate in sesame plants, could explain the associated membrane damage that affects photosynthetic efficiency in dry conditions. Consequently, negative impacts on vegetative growth parameters would be encountered. The fresh weights, shoot and root lengths of *Ephedra foliate* and *Abelmoschus esculentus* plants were discovered to be reduced by drought stress compared to the well-watered ones, as reported by Al-Arjani et al. (2020) and Jabborova et al. (2021). Begum et al. (2019) and Abdelhameed et al. (2024) also documented a significant decline in total carotenoids and chlorophyll (Chl.), which was followed by a decline in the growth attributes of maize and malva plants.

One of the numerous methods for addressing drought-prone stress in plants is the exploitation of indigenous microorganisms. These advantageous microorganisms may be employed in either the direct or indirect manner to enhance mineral fertilization and tolerance mechanisms. Arbuscular mycorrhizae fungi (AMF) possess the ability to improve crop productivity and soil condition in environmentally conscious farming practices, particularly in soils that are deficient in nutrients **(**Abdelhameed and Metwally 2022; Rasouli et al. 2023**)**. AMF are substantial groups of soil-based microorganisms that establish a mutually beneficial relationship with a diverse array of plants **(**Brundrett and Tedersoo 2018; Metwally and Abdelhameed 2019; Abdelhameed and Metwally 2019**)**. A glomalin-related soil protein is exuded by these fungi, thereby improving the capture of carbon out of the soil and contributes to the soil aggregate's stability **(**Driver et al. 2005; Yang et al. 2017; Matos et al. 2022**)**. Moreover, AMF are linked to soil properties that enhance porosity, which promotes improved nutrient uptake and enhances the plant-water relationship **(**Ingraffia et al. 2019; Badr et al. 2020**)**. Several methods, including the mitigation of ROS and oxidative damage and the enhancement of antioxidant defense systems that are enzymatic and non-enzymatic are used by AMF to control plant development in both biotic and abiotic environments **(**Abd-Elghany et al. 2021; Spinoso-Castillo et al. 2023; Abdelhameed and Metwally 2024**)**.

Most specifically, under drought stress, AMF induce plant tolerance through maintaining the level of hormones (Sheteiwy et al. 2021a) and enhancing soil enzyme activities (Sheteiwy et al. 2021b; Nader et al. 2024). As well, AMF is known to have apparent tool to strengthen the host plants' development, uptake of nutrients, and water in drought-stricken areas **(**Hashem et al. 2019; Al-Arjani et al. 2020; Jabborova et al. 2021; Li et al. 2022; Nader et al. 2024**)**. Additionally, it has been shown to improve the efficiency of water utilization and stomatal conductance (Birhane et al. 2012; Hashem et al. 2016**)**. Oliveira et al. (2022) showed that drought reduced the efficiency of photosynthesis, stomatal conducting capacity, and transpiration rate in soybean cultivars. However, AMF-inoculated plants showed increased values for these photosynthetic characteristics than non-inoculated plants.

Osmo-protectants, such as polyamines (PAs), are believed to safeguard plants from harmful environmental conditions, including drought and salinity, in different crops **(**Tyagi et al. 2022; Wang et al. 2023**)**. Plants consume ornithine and arginine as precursors for the manufacture of polyamines (PA) through ornithine decarboxylase (ODC) and arginine decarboxylase (ADC) enzymes, which convert into the amino acid putrescine (Put). The higher polyamines; spermidine (Spd) and spermine (Spm) are subsequently produced from putrescine (Put) by the enzymes known as spermidine synthase (SPDS) and spermine synthase (SPS), respectively. Amino propyl groups are produced when S-adenosylmethionine decarboxylase (SAMDC) decarboxylates S-adenosylmethionine (SAM), and these groups are subsequently added to Put and Spd for the production of Spd and Spm polyamines, respectively **(**Chen et al. 2019**)**. Polyamines (PAs), whether applied externally or produced internally by genetic modification, are recognized as potent bio-stimulants that can improve plant growth, productivity, and stress resistance **(**Biondi et al. 2022; Tyagi et al. 2022**)**. Genetically modified plants to increase the expression of SAMDC or ADC, SPDS, or SAMDC genes have the capacity to withstand a range of stressors, such as drought, hot and low temperatures **(**Kasukabe et al. 2004; Luo et al. 2017; Marco et al. 2019**)**. In a variety of plant species, including wheat, the external application of PAs has been demonstrated to substantially alleviate drought stress symptoms **(**Gupta et al. 2012; Gholizadeh et al. 2022; Wasaya et al. 2022; Li et al. 2024**)**, finger millet **(**Satish et al. 2018**)**, *Rosa damascene* **(**Hassan et al. 2018**)**, lettuce **(**Zhu et al. 2019**)**, tomato **(**Upadhyay et al. 2021**)**, soybean **(**Dawood and Abeed 2020**)**, winterberry **(**Xie et al. 2023**)**. Positively charged PAs are capable of forming bonds with negatively charged macromolecules within the cell, including nucleic acids, proteins, and phospholipids. These associations serve as intermediaries for the modifications in the chemical or physical characteristics of macromolecules, which regulate their biological activities directly. Environmental stress has been demonstrated to be mitigated by the acid-neutralizing, reduction of ROS by detoxifying enzymes, antioxidant, membrane and cell wall-stabilizing and induction of molecular chaperone; properties of PAs when applied externally to plants. Hormones, calcium, and nitric oxide are just a few of the signaling molecules that PAs interact to control the growth and stress tolerance of plants. Hence, they are perceived as mediators for plant stress **(**Montilla-Bascón et al. 2017; Biondi et al. 2022; Tyagi et al. 2022; Li et al. 2024**)**.

The goal of the current study's experiment was to evaluate how mutualistic interactions with AMF enable soybean plants to withstand morphological and physio-biochemical stress. Studies regarding the reaction of plant polyamines to the mutualistic relationship between AMF and challenging environmental circumstances are scarce, despite the advantageous association of AMF with plants. Therefore, after AMF colonization, the present work's objective is also to clarify how the genes involved in PAs production respond in soybeans with different drought stress resilience. Most specifically, the PAs biosynthesis genes were in silico characterized and relative gene expression of three of them [Ornithine decarboxylase (ODC2), Spermidine synthase (SPDS) and Spermine synthase (SpS)] were quantified in response to different treatments; i.e. drought and/or AMF inoculation.

## Materials and methods

### Arbuscular mycorrhizal fungi (AMF) and trap culture method

The AMF; *Gigaspora margarita, Funneliformis mosseae*, *Rhizophagus irregularis,* and *F. constrictum* were formerly extracted from the rhizosphere of plants in Minia Al-Qamh, El-Sharkia Governorate, Egypt, through wet sieving and decanting procedures in accordance to Gerdemann and Nicolson (1963). To summarize, 500 g of rhizospheric soil was placed in a container, then 10 L of water was added and thoroughly mixed to create a soil–water suspension. To extract spores, the suspension was sieved via sieves with pore sizes of 500, 250, 150, and 38 µm after settling for 5 min to remove insoluble as well as heavy particles. The extracted AMF spores were identified using the features of asexual spore structures described in a manual available by INVAM (2014). The AMF spore mix was grown for 6 months on Sudan grass (*Sorghum sudanenses* Pers.) roots in a sterilized substrate of sandy clay soil as trap plants to expand the AMF population **(**Stutz and Morton 1996**)**.

### Soybean seeds and soil

Soybean seeds (*Glycine max* L.) var. Giza 111 were purchased from Egypt's Agricultural Research Centre in Giza. Morphologically identical seeds were surface disinfected with sodium hypochlorite (5%) for 10 min and then rinsed with sterilized dist. water. The experimental soil employed in the current study was taken from Minia Al-Qamh in El-Sharkia Governorate and has the following parameters (%): clay: silt: sand, 62.8: 27.5: 9.7; moisture content, 3.15; organic matter, 1.93; and total phosphorus, 0.69. The soil's pH was 7.68. The soil was autoclaved for 3 h at 121 °C, then cooled and divided into plastic pots.

### Plant growth condition and drought stress application

Soybeans were propagated in disinfected plastic pots (25 cm diam.) filled with 2 kg sterilized soil under greenhouse conditions in the plant growth chamber of the Botany and Microbiology Department, Faculty of Science, Zagazig University, Egypt (Latitude: 30.5872° N and Longitude: 31.5036° E), with a 14-h light and 10-h dark photocycle at 27 °C. The relative humidity had been adjusted to 60%. One week later, the propagated seeds were thinned to 5 per pot. Soybean plants were divided into two groups: non-AMF, in which soybean seeds were planted in pots with an additional 50 g of sterilized soil per pot, and AMF, in which soybean seedlings received 50 g of AMF inoculum per pot (approx. 70 spores/g trap soil and infected root pieces, M = 75%) at the sowing date. Both categories (non-AMF and AMF-inoculated) were watered for the first 20 days of growth at 90% field capacity. To apply drought, each plant group was divided into three sets, resulting in a fully randomized experiment with a 2 × 3 factorial plan. Each set contained five replicates (n = 5). Drought stress was applied over a 20-day period using three distinct water irrigation regimes (severe: 25% FC, mild: 50% FC, and full: 90% FC) based on Piper (1947). Tap water was used in irrigation.

### Measurements

#### Calculating the extent of colonization by arbuscular mycorrhizae

Soybean roots were wisely cleaned in chilled water (4 °C) to eliminate dirt particles. Fine roots were collected, sliced into 1 cm lengths, and stored in a solution of formalin/acetic acid/ethyl alcohol (5:5:90)(v/v/v) up to extra treating. Root segments were then cleared with KOH (10%) at 90 °C, immersed in a 7.5% hydrogen peroxide (H_2_O_2_) solution for 5 min at ambient temperature, suspended in 1N HCl, and dyed with trypan blue (0.05%)-lactophenol **(**Phillips and Hayman 1970**)**. Dyed root segments were put on lactophenol-coated glass slides then inspected under a light microscope at 100 magnifications to determine AMF colonization of the roots. Fungal infection (mycelium, arbuscules, and vesicles) plus development were seen in the inoculated soybean roots. The Trouvelot et al. (1986) approach was among the most commonly used methods for AMF colonization assessment and was used to assess the frequency (F%), intensity (M%), and rate (A%) of mycorrhizal colonization in the stained roots by means of the Mycocalc software (http://www.dijon-inra.fr/mycocalcprg).

### Plant sampling and determination of morphological parameters

Following the period of drought stress cycle, the soybean plants of 40 days-old from each treatment were collected, their shoots and roots were assessed individually to measure their fresh (Fwt) and dry weights (Dwt). The shoot besides root Dwt were evaluated after freshly harvested samples are kept at 60 °C for 72 h. Root lengths, shoot heights, and root: shoot ratio (R/S) were measured. Moreover, the soybean's mycorrhizal dependence (MD) was measured with the following equation (Menge et al. 1978):

$$\frac{\text{R}}{\text{S }}\text{ ratio }({\text{\%}}) = \frac{(\text{ Root Dwt of plants })}{(\text{ Shoot Dwt of plants })} \times 100$$

$$\text{Mycorrhizal dependency }(\text{MD}) = \frac{(\text{ Total biomass of plants without AMF })}{(\text{ Total biomass of plants inoculated with AMF})} \times 100$$

### Plant physio-biochemical constituents

#### Photosynthetic traits and gas exchange parameters

Metzner et al. (1965) technique was used to assess the chlorophyll a, b, and carotenoids in leaf tissues (0.5 g) that were extracted in 85% acetone. The extract's absorbance was taken at 452.5, 644, and 663 nm against acetone as a blank by a spectrophotometer (UV-visible spectrophotometer, Model Ultra-3660) next centrifugation at 3000 rpm for 5 minutes. The chlorophyll a, b, and carotenoids were then calculated in mg g^−1^ Fwt.

Gas exchange characteristics were measured in the Ecology Laboratory, Faculty of Science, Helwan University on a bright day under ambient CO_2_ and humidity conditions using an LC pro-SD infrared gas analyzer (IRGA) from ADC BioScientific Ltd. in the UK. Parameters measured include leaf stomatal conductance (*gs*, mmol H_2_O m^−2^ s^−1^), transpiration rate (*E*, mmol H_2_O m^−2^ s^−1^), net photosynthetic rate (*A*, μmol CO_2_ m^−2^ s^−1^), intrinsic water use efficiency (WUEi) (*A/gs*, µmol CO_2_ mmol^−1^ H_2_O), besides CO_2_ concentration ratio (*ci/ca*) between internal and atmospheric sources.

### Relative water content (RWC) and membrane stability index (MSI) assessment

The RWC was assessed by the Barr and Weatherley (1962) procedure. The turgid weight (Twt) was evaluated by submerging the leaves in water at 4 °C in the dark for 24 h. The RWC was calculated with the following formula:

$${\text{RWC }}\left( {\text{\% }} \right){ } = \frac{{\left( {{\text{Fwt - }}\,{\text{Dwt}}} \right){ }}}{{{ }\left( {{\text{Twt}}\,{\text{ - Dwt}}} \right){ }}}{\text{ X }}100$$

where; Fwt: fresh weight, Dwt: dry weight, Twt: turgid weight.

To assess the MSI of soybeans under both controlled and drought-stressed environments, the Hayat et al. (2010) technique was employed. In brief, four leaves from each treatment were gathered, diced (100 mm^2^ leaf discs), and placed in two tubes, each with 30 mL of double-distilled water. The first set of samples was incubated at 25 °C with shaking for 4 h before being measured for electrical conductivity (EC_1_) using an Oakton PC2700 conductivity meter (Oakton Instruments, USA), which quantifies membrane damage by measuring EL. The second set was autoclaved at 121 °C for 20 min, cooled to 25 °C, and its conductivity (EC_2_) was assessed. The MSI was determined using the following formula:

$${\text{MSI}} = \left[ {{1} - \left( {{\text{EC}}_{{1}} /{\text{EC}}_{{2}} } \right)} \right] \times {1}00$$

### Oxidative stress markers (electrolyte leakage, lipid peroxidation and H_2_O_2_ assay)

Sullivan's (1979) formula was used to calculate electrolyte leakage (EL) in a solution of twenty leaf discs (0.5 cm) at ambient temperature, 45–55 °C for 30 min, and 100 °C for 10 min in order to record EC_a_, EC_b_, and EC_c_; respectively. The formula was as follows:

$${\text{EL }}(\%)= [({{\text{EC}}_{\rm b}}-{{\text{EC}}_{\rm a}})/{{\text{EC}}_{\rm c}}]\times 100$$

Malondialdehyde (MDA) levels in soybean leaves, which indicate membrane lipid peroxidation, were measured in μmol g^−1^ Fwt **(**Ohkawa et al. 1979**)**. Fresh leaf tissue (500 mg) was macerated in 2-thiobarbituric acid (TBA; 0.5%) and trichloroacetic acid (TCA; 20%). Subsequently heated at 95 °C for 30 min, the mixture was rapidly chilled and centrifuged for 10 min at 6000 rpm. The absorbance was recorded at 532 nm besides corrected for non-specific turbidity via subtracting the absorbance at 600 nm.

The H_2_O_2_ content of leaf samples (500 mg) was evaluated **(**Alexieva et al. 2001**)** following homogenization in 5 mL of 0.1% TCA and centrifugation for 20 min. Afterward, 0.5 mL of supernatant was added to 0.5 mL of 10 mM potassium phosphate buffer (pH 7.0) besides 1 mL of 1 M potassium iodide. The absorbance was registered at 390 nm, and the H_2_O_2_ concentration was displayed.

### Assay of protective molecules content and total antioxidant capacity (TAC)

After homogenizing fresh soybean leaves in 10 mL of 50 mM potassium phosphate buffer (pH 7.0), the total protein content in mg/g Fwt was calculated and measured at the absorbance of 700 nm **(**Lowry et al. 1951**)**, with bovine serum albumin as a standard. The proline level was determined using the method given by Bates et al. (1973). The glycine betaine content of lyophilized fresh soybean leaves was determined using a colorimetric method **(**Grieve and Grattan 1983**)**. Combine 1.5 mL of 2 N H_2_SO_4_ with lyophilized tissue, heat in a water bath at 60 °C for 10 min, then combine with 50 µL cold KI-I_2_. Samples were centrifuged for 15 min at 4 °C after being stored at 0–4 °C for 16 h, and they were then left on ice for an hour. After gathering the supernatant, 1,2-dichloroethane (4.5 mL) was added. After two hours of room temperature incubation, the mixture's absorbance at 365 nm was calculated. TAC in methanolic soybean extracts was measured using ammonium molybdate reagent **(**Prieto et al. 1999**)**. After 90 min of incubation in a boiling water bath, the tubes were cooled and the absorbance at 695 nm was measured. The TAC was given in mg of comparable ascorbic acid per g of Fwt.

### Activity of alkaline (ALP) and acid phosphatase (ACP)

Using the Tabatabai and Bremner method (1969) and the *p*-nitrophenyl phosphate disodium salt (*p*NPP) technique, the activity of enzymes (ACP and ALP) in soybean leaves was determined. For the ACP activity reaction, leaf tissues were crushed in 5 mL of ice-cold sodium acetate buffer (0.1 M, pH 5.0), or for the ALP activity, in sodium phosphate buffer (100 mM, pH 8.0). The resultant homogenate was centrifuged for 15 min. The appropriate homogenizing buffers (pH 5.0 or pH = 8.0 sodium acetate buffer) were used to incubate the supernatant. The enzyme hydrolyzed 5 mM *p*NPP as a substrate to produce *p*NP. The activity was measured colorimetry using spectrophotometer at 410 nm and expressed as μmol of *p*NP released per minute.

#### Easily and totally extracted glomalin

To find the concentration of total extractable glomalin-related soil proteins (TE-GRSP) and soil easily extractable (EE-GRSP) in the rhizosphere soil, soil samples were taken **(**Wright and Upadhyaya 1996**)**. In order to assess the EE-GRSP, 2 g of soil samples were combined with 8 mL of 20 mM sodium citrate (pH 7.0), autoclaved for 30 min at 121 °C, and then centrifuged for 15 min at 6000 rpm. The supernatant was stored at 4 °C for future analysis. A 2 g soil sample was autoclaved for 60 min together with 8 mL of 50 mM sodium citrate (pH 8.0) and centrifuged for 15 min at 6000 rpm as part of the TE-GRSP extraction procedure. Bradford (1976) assessed the contents using bovine serum albumin as a standard.

#### Molecular analysis of polyamines (PAs) biosynthesis genes

The PAs biosynthesis genes were in silico characterized and relative gene expression of three of them were quantified in response to different treatments; i.e. drought and/or AMF inoculation.

### In silico genomics analysis for PAs biosynthesis genes in soybean

To determine the number of possible genes that may be involved in soybean PAB. The *G. max* genome database was screened for the possible targets using reference sequence of *G. max* deposited in NCBI GeneBank (Assembly: *Glycine_max*_v4.0 (GCF_000004515.6) cultivar Williams 82, taxon:3847) (https://www.ncbi.nlm.nih.gov/datasets/genome/GCF_000004515.6/). All genomic basic information including genes, transcript and protein IDs, their locations, sequences length, and the chromosomal localization was collected and tabulated in Table 6. The retrieved transcript sequences were used as a template to design the gene specific primers used in the gene expression analysis (Suppl. Table 1) using primer designing tool (https://www.ncbi.nlm.nih.gov/tools/primer-blast/).

### Quantitative gene expression analysis of PAs biosynthesis genes

#### RNA extraction, DNase treatment and cDNA preparation

Soybean leaf tissues were collected from each group and finely ground in liquid nitrogen then the total RNA was extracted using Zymo Direct-Zol RNA Miniprep kit (Zymo Research, R2050) according to manufacturer’s instruction. The isolated RNA was DNase treated to remove any DNA contaminant using Life Technologies'-Invitrogen PureLink^®^ DNase Kit (cat. # 12185010). Successful DNase treatment was confirmed by the absence of any PCR product after 39 cycles using Actin6 gene primers and DNase treated RNA as a template. SuperScriptTM II reverse transcriptase was used to transcribe cDNA from 2 µg of RNA that had been treated with DNase with oligo (dt)18 primers (cat.# 18064–014, Life technologies-Invitrogen), following the manufacturer’s instructions.

### Quantitative PCR (qPCR)

Cycling settings were performed using a QuantStudio 5 Real-Time PCR System (Applied Biosystems, USA) and HERA PLUS SYBR green qPCR master mix (cat. # 600,882, Agilent Technologies-Stratagene) as follow: 95 °C (2 min) then 39 cycles of 95 °C (15 s), and 60 °C (20 s). Each reaction was performed in duplicate using GmActin6 as an internal control **(**Hu et al. 2009**)**. The gene IDs and primer sequences that were used were listed in Supplementary Table 1. The differences in expression were assessed by the comparative Ct method using ΔΔCt according to Livak and Schmittgen (2001).

### Statistical analyses

The morpho-physiological besides biochemical parameters of soybean under drought stress were affected by AMF addition and the acquired results were statistically analyzed by software of statistical package for the social sciences (SPSS 14.0 windows evaluation version) **(**Spiegel 1975**)**. The data was compared using one-way analysis of variance (ANOVA). The graphical presentation was created using Origin. Pearson correlation coefficients were produced for soybean's measured variables. Hierarchical clustering analysis and principal component analysis (PCA) graphs between different treatment used in this study and soybean measured parameters were created using Past.

## Results

### Morphological parameters

**Fig. 1:**
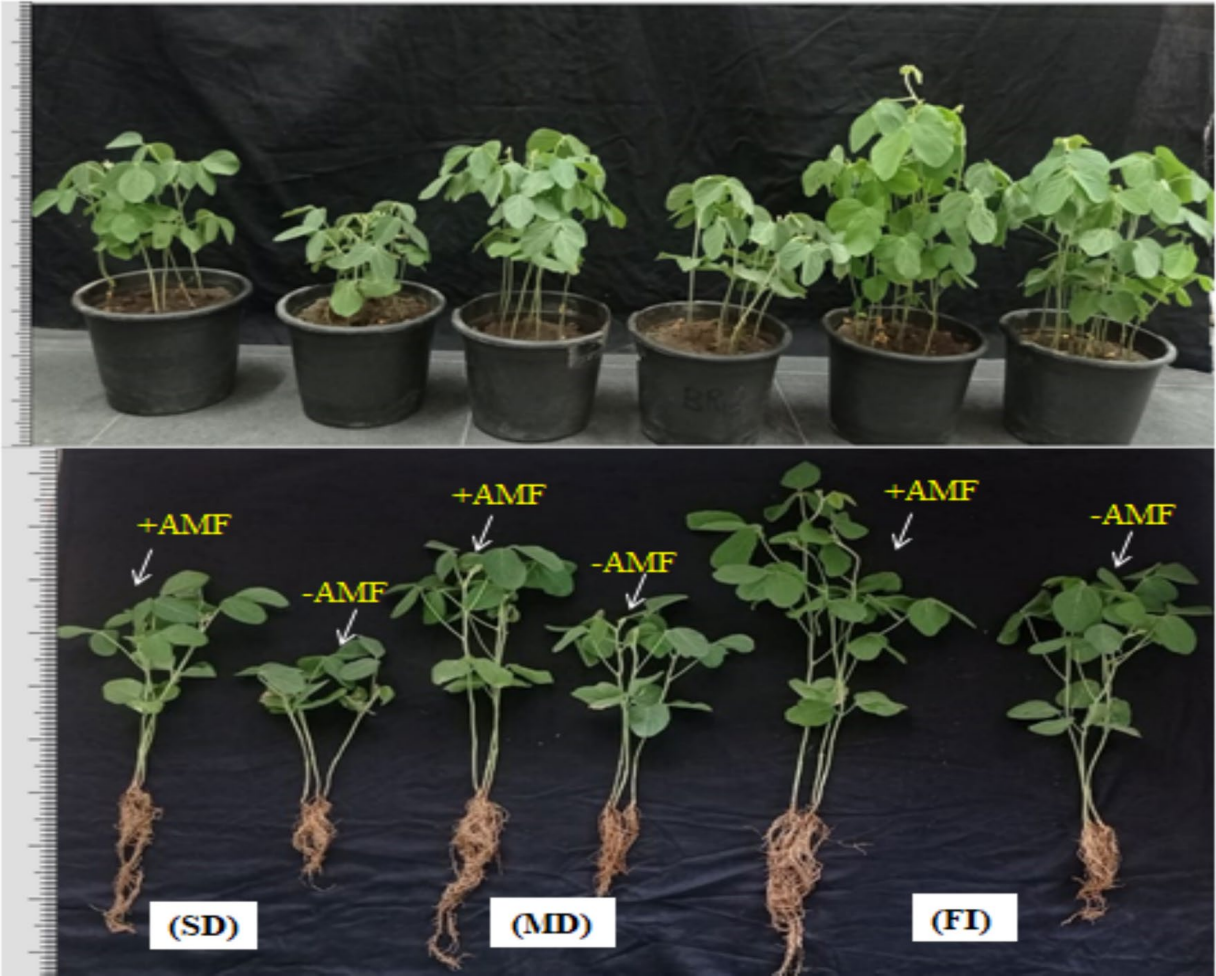
Effect of drought stress and AMF colonization on the morphological appearance (shoot and root) of soybean under three irrigation regimes; FI full irrigation, MD mild drought, and SD severe drought

Figure 1 and Table 1 indicated that exposing non-AMF and AMF-inoculated soybean plants to both severe (25% FC) and mild (50% FC) irrigation water regimes occasioned in a major reduction of all morphological attributes such as root and shoot length, root and shoot biomass, and that severe drought produced a significant diminution in these parameters when compared to mild drought. Furthermore, (Fig. 1) depicts the effects of drought stress as well as AMF colonization on soybean morphological appearance (shoots and roots). The extreme drought lowered root length and shoot height by 29.26 and 44.99%, respectively, compared to non-drought soybean plants (90% FC). Nevertheless, AMF-inoculated plants outperformed non-AMF plants in both drought and well-watered conditions. AMF inoculation raised soybean plant shoot height, shoot Fwts and Dwts, and R/S ratio by 31.76, 28.88, 33.11, and 17.17%, respectively, besides root length, root fresh and dry weight by 20.68, 23.39, and 56.03%, respectively, in contrast to untreated plants facing extreme drought stress.

**Table 1:** Impact of AMF inoculation on growth traits of soybean grown under three irrigation regimes

Irrigation regime	AMF	Shoot height (cm/plant)	Root length (cm/plant)	Shoot weight (g/plant)		Root weight (g/plant)		No. leaves/ plant
				Fresh	Dry	Fresh	Dry	
FI	–	26.67 ± 0.705b	13.67 ± 0.361b	2.86 ± 0.075b	0.5868 ± 0.0155b	0.715 ± 0.0189c	0.0926 ± 0.0024c	5.33 ± 0.141b
	+	37.00 ± 0.978a	18.33 ± 0.484a	3.79 ± 0.100a	0.7294 ± 0.0192a	1.580 ± 0.0418a	0.1739 ± 0.0046a	6.00 ± 0.158a
MD	–	22.00 ± 0.582c	10.67 ± 0.282 cd	2.07 ± 0.054d	0.4705 ± 0.0124c	0.601 ± 0.0158d	0.0881 ± 0.0023c	4.33 ± 0.114de
	+	25.67 ± 0.678b	13.33 ± 0.352b	2.63 ± 0.069c	0.5637 ± 0.0149b	1.053 ± 0.0278b	0.1555 ± 0.0041b	5.00 ± 0.132bc
SD	–	14.67 ± 0.387e	9.67 ± 0.255d	1.35 ± 0.035f	0.3110 ± 0.0082e	0.359 ± 0.0094f	0.0605 ± 0.0016d	4.00 ± 0.105e
	+	19.33 ± 0.511d	11.67 ± 0.308c	1.74 ± 0.045e	0.4142 ± 0.0109d	0.443 ± 0.0117e	0.0944 ± 0.0024c	4.67 ± 0.123 cd

Data presented represent the mean of 5 replicates with standard error. Different letters indicate significant differences among treatments using a one-way ANOVA followed by the Duncan’s multiple range test (*p* < 0.05)

FI full irrigation, MD mild drought, and SD severe drought

#### AMF colonization of soybean roots

**Fig. 2:**
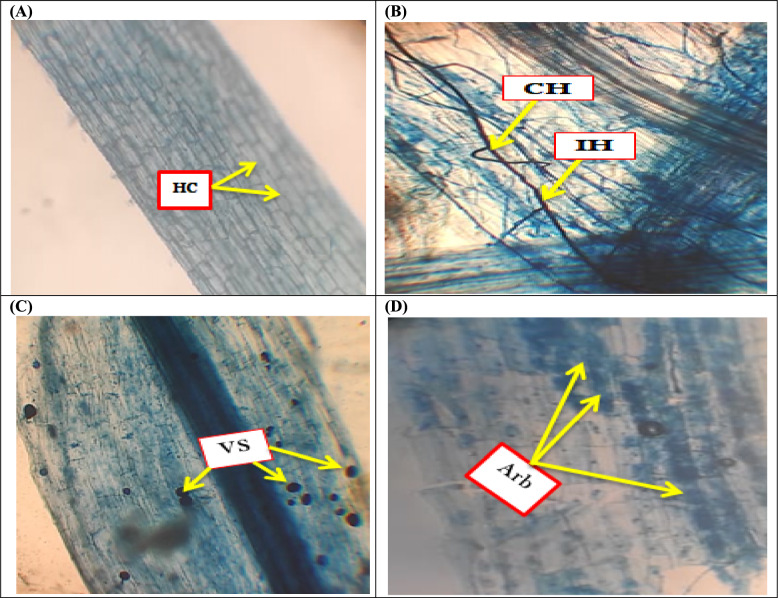
AMF colonization of soybean 20 days after drought application; **A** Control treatment and (**B-D**) Photomicrographs of AMF-inoculated soybean plant roots illustrating different AMF structural components. Arb Arbuscule, IH Intraradical hyphea, VS Vesicles, CH Coiled hyphae, HC Host cell

To confirm that the change in growth characteristics was caused by AMF inoculation, AMF colonization rates were measured. Table 2 shows the extent of AMF colonization in soybean plant roots. In neither well-irrigated nor drought-stressed environments did non-AMF infected plants exhibit mycorrhizal colonization (Fig. 2A). Microscopic photographs of soybean root pieces were also traced to determine the existence of various AMF morphological structures, as well as images of AMF organs that appear in blue stain, such as vesicles, intraradical hyphae, coiled hyphae, and arbuscules (Fig. 2B-D). Soybean roots underwent a considerable reduction in the rate of AMF colonization during both mild and severe drought stress, as demonstrated by lower levels of mycelium, vesicles, and arbuscules compared to well-watered AMF-inoculated plants (Table 2). Severe drought stress lowered the percentages of F, M, and A by 14.27, 36.60, and 47.22 percent, respectively, when compared to full water irrigation circumstances.

**Table 2:** Impact of drought stress on the AMF colonization potential rates, R/S ratio and mycorrhizal dependency (MD) (%) of soybean grown under three irrigation regimes

Irrigation regime	AMF	F (%)	M (%)	A (%)	R/S Ratio (%)	Mycorrhizal dependency (%)
FI	–	– – – – –	– – – – –	– – – – –	15.785 ± 0.417d	– – – – –
	+	95.45 ± 2.525a	49.91 ± 1.320a	40.93 ± 1.082a	23.850 ± 0.630b	66.579
MD	–	– – – – –	– – – – –	– – – – –	18.736 ± 0.495c	–
	+	86.36 ± 2.284b	37.64 ± 0.995b	30.80 ± 0.814b	27.596 ± 0.730a	72.692
SD	–	– – – – –	– – – – –	– – – – –	19.449 ± 0.514c	– – – – –
	+	81.82 ± 2.164b	31.64 ± 0.837c	21.60 ± 0.571c	22.789 ± 0.602b	78.186

F% frequency of colonization, M% colonization intensity as well as A%: the level of arbuscular development. Data presented represent the mean of 5 replicates with standard error. Different letters indicate significant differences among treatments using a one-way ANOVA followed by the Duncan’s multiple range test (*p* < 0.05)

FI full irrigation, MD mild drought, and SD severe drought

Although AMF colonization decreased with increasing drought stress, mycorrhizal dependence (MD) augmented in drought stressed soybean as compared to those under full water irrigation. MD was 72.69% in mild drought and 78.18% in severe drought for total Fwt.

#### Photosynthetic traits and gas exchange parameters

**Fig. 3:**
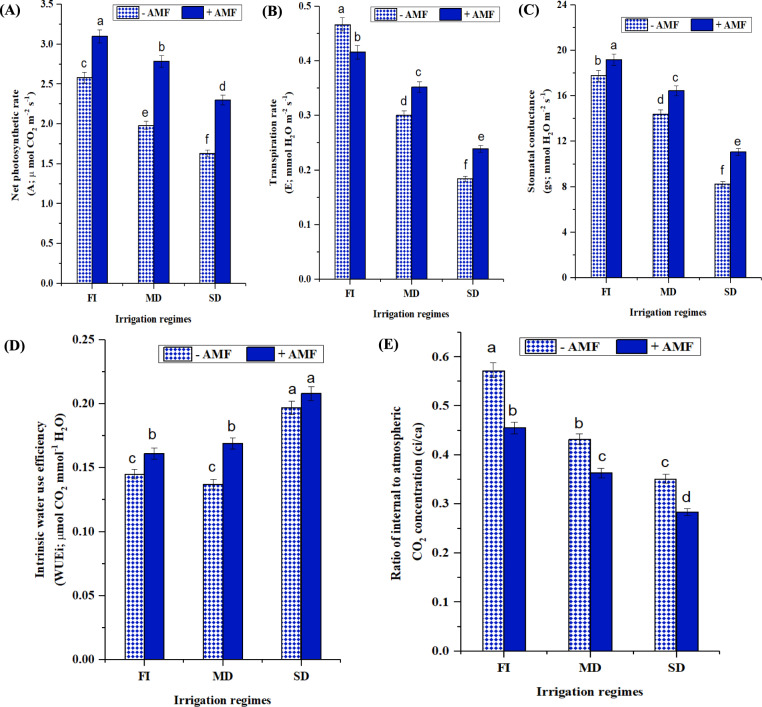
Impact of AMF inoculation on gas exchange parameters; **A** net photosynthetic rate (*A*), μmol CO_2_ m^−2^ s^−1^), **B** transpiration rate (*E*, mmol H_2_O m^−2^ s^−1^), **C** stomatal conductance (*gs*, mmol H_2_O m^−2^ s^−1^), **D** intrinsic water use efficiency (*WUE*) (*A/gs*, µmol CO_2_ mmol.^−1^ H_2_O) and **E** ratio of internal to atmospheric CO_2_ concentration (*ci/ca*) of soybean grown under three irrigation regimes; full irrigation (FI), mild drought (MD), and severe drought (SD). Data presented represent the mean of 5 replicates with standard error. Different letters indicate significant differences among treatments using a one-way ANOVA followed by the Duncan’s multiple range test (*p* < 0.05)

In contrast to the treatment that is well-watered, the mild and severe droughts had continuously decreasing values for the examined photosynthetic and gas exchange characteristics (Table 3 and Fig. 3A-E). Soybean plant total pigment, *E*, *A*, and *gs* levels were considerably reduced under severe drought stress by 15, 36.79, 60.59, and 53.50%, respectively, compared to non-stressed conditions. In contrast, the addition of AMF inoculum significantly improved all observed photosynthetic parameters. Under non-stressed conditions, AMF-inoculated soybean plants had higher levels of photosynthetic pigments and gas exchange parameters than non-AMF soybean plants. The percentage increase for *gs* was 8%, 20% for *A*, and 11% for *A*/*gs* (Fig. 3). Under extreme drought stress, AMF boosted *A*,* E*, and *gs* levels by 40.87, 29.89, and 33.65%, respectively, as compared to non-AMF plants growing in well-watered circumstances. Water use efficiency (WUE) rose dramatically under drought stress or after AMF inoculation. Nonetheless, their applications resulted in a lower Ci/Ca ratio. Besides, the decreased Chl. content in soybean plants under drought stress and the capacity of AMF to stop this depletion was observed.

**Table 3:** Impact of AMF inoculation on pigment fractions (mg/g Fwt) of soybean grown under three irrigation regimes

Irrigation regime	AMF	Chl a (mg/g Fwt)	Chl b (mg/g Fwt)	Total Chl (mg/g Fwt)	Carotenoids (mg/g Fwt)	Total pigment (mg/g Fwt)
FI	–	2.645 ± 0.0699ab	1.207 ± 0.0319b	3.852 ± 0.101ab	1.820 ± 0.048ab	5.672 ± 0.150ab
	+	2.763 ± 0.0731a	1.327 ± 0.0351a	4.090 ± 0.108a	1.894 ± 0.050a	5.983 ± 0.158a
MD	–	2.486 ± 0.0657bc	0.999 ± 0.0264c	3.486 ± 0.092c	1.635 ± 0.043 cd	5.121 ± 0.135c
	+	2.528 ± 0.0668bc	1.032 ± 0.0273c	3.560 ± 0.094bc	1.696 ± 0.044bc	5.257 ± 0.139bc
SD	–	2.371 ± 0.0627c	0.950 ± 0.0251c	3.321 ± 0.087c	1.500 ± 0.039d	4.821 ± 0.127c
	+	2.406 ± 0.0636c	0.977 ± 0.0258c	3.384 ± 0.089c	1.593 ± 0.042 cd	4.976 ± 0.131c

Values are means of 5 replicates with standard error. Different letters indicate significant differences among treatments using a one-way ANOVA followed by the Duncan’s multiple range test (*p* < 0.05)

FI full irrigation, MD mild drought, and SD severe drought

#### Relative water content and membrane stability index

**Fig. 4:**
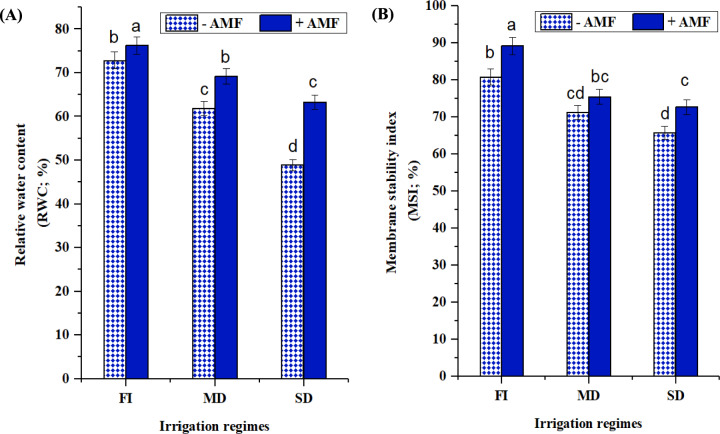
Impact of AMF inoculation on (**A**): relative water content and (**B**): membrane stability index of soybean grown under three irrigation regimes; full irrigation (FI), mild drought (MD), and severe drought (SD). Data presented represent the mean of 5 replicates with standard error. Different letters indicate significant differences among treatments using a one-way ANOVA followed by the Duncan’s multiple range test (*p* < 0.05)

When soybean leaves were subjected to mild or severe drought as opposed to full water irrigation, their RWC and MSI drastically dropped (Fig. 4A and B). RWC and MSI reductions were more apparent under extreme drought stress, with values of 32.75 and 18.65% lower than non-stressed plants, respectively. This effect was diminished in the AMF plants, which had RWC values analogous to their non-drought exposed counterparts (Fig. 4A). A comparable effect was reported for MSI, which remained substantially high in non-stressed plants regardless of AMF status (Fig. 4B). AMF colonization improved the RWC and MSI parameters by 22.67 and 10.52% compared to non-treated plants under severe drought stress circumstances.

#### Oxidative stress markers (EL, lipid peroxidation and H_2_O_2_)

**Fig. 5:**
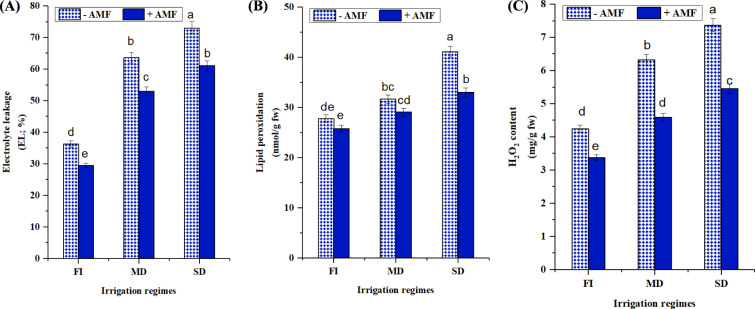
Impact of AMF inoculation on oxidative stress markers; **A** electrolyte leakage (EL; %), **B** lipid peroxidation; (MDA) (nmol/g Fwt) and (**C**): H_2_O_2_ content (mg/g Fwt) of soybean grown under three irrigation regimes; full irrigation (FI), mild drought (MD), and severe drought (SD). Data presented represent the mean of 5 replicates with standard error. Different letters indicate significant differences among treatments using a one-way ANOVA followed by the Duncan’s multiple range test (*p* < 0.05)

To understand more about the reasons for the growth differential between well-irrigated and drought-stressed soybean plants, the level of oxidative stress was investigated. This was accomplished by measuring lipid peroxidation (MDA level), ion leakage (EL), and ROS formation (H_2_O_2_ concentration) in soybean leaves under water shortage conditions. The results displayed that all levels of oxidative stress markers steadily rose with decreased irrigation regime, with the highest values obtained during extreme drought stress, as shown in (Fig. 5A-C). In well-watered conditions, The MDA levels of plants injected with AMF and those that were not showed any significant differences (Fig. 5B). The mild irrigation regime significantly increased the accumulation of EL, MDA, and H_2_O_2_ in soybean leaves (75.39, 13.80, and 49.21%) more obviously in non-AMF inoculated plants. In contrast, AMF application resulted in a significant drop in El, MDA, and H_2_O_2_, reaching 16.50, 19.53, and 25.94%, respectively, under extremely severe drought circumstances, in comparison to the similar controls.

#### Protective molecules and total antioxidant capacity (TAC)

As compared to well-watered conditions, the data displayed in Table 4 exhibited that the levels of osmo-protectants (free proline and glycine-betaine) in soybean plant leaves under severe water deficit conditions were significantly greater, with the highest values obtained for AMF-treated plants. Conversely, however, as compared to plants that received regular irrigation, the soluble protein content of soybean plant leaves was unaffected by drought stress. Severe drought stress boosted proline plus the amounts of glycine-betaine by 104.61 and 50.44%, respectively, when compared to plants that received adequate water. Furthermore, under extreme drought stress, AMF colonization increased proline, protein, and glycine-betaine levels (by 34.41, 34.30, and 17.85%, respectively) compared to the non-AMF inoculated group. Plants without AMF injections had the lowest proline and glycine-betaine levels under well-watered circumstances. The data displayed in Table (4) demonstrated that drought stress and AMF application altered soybean plant TAC. Water stress induced the greatest increase in TAC. Notably, soybean grown under extreme drought stress and injected with AMF had the highest TAC, increasing by 33.86% compared to the control. Soybean plants cultivated under well-irrigated circumstances and AMF-inoculated showed negligible TAC.

**Table 4:** Impact of AMF inoculation on osmo-protectants and total antioxidant capacity (TAC) of soybean grown under three irrigation regimes

Irrigation regime	AMF	Protein content (mg/g Fwt)	Proline content (µmols/g Fwt)	Glycine betaine (mg/g Fwt)	TAC (mg/g Fwt)
FI	–	15.261 ± 0.403b	4.663 ± 0.123e	2.721 ± 0.072d	2.681 ± 0.070e
	+	21.111 ± 0.558a	5.209 ± 0.137de	2.814 ± 0.074d	3.081 ± 0.081d
MD	–	14.511 ± 0.383b	5.845 ± 0.154d	3.602 ± 0.095c	3.443 ± 0.091bc
	+	22.191 ± 0.587a	6.815 ± 0.180c	4.035 ± 0.106b	3.889 ± 0.102a
SD	–	15.951 ± 0.422b	9.540 ± 0.252b	4.092 ± 0.108b	3.266 ± 0.086 cd
	+	21.441 ± 0.567a	12.815 ± 0.339a	4.822 ± 0.127a	3.589 ± 0.094b

Data presented represent the mean of 5 replicates with standard error. Different letters indicate significant differences among treatments using a one-way ANOVA followed by the Duncan’s multiple range test (*p* < 0.05)

FI full irrigation, MD mild drought, and SD severe drought

#### Phosphatase enzyme activities (ACP and ALP)

ACP and ALP activities were dramatically increased in soybean plants infected with AMF, but were significantly reduced by drought stress. The activity of ACP was found to be exceeding ALP's level. In plants that receive enough water and infected with AMF, ACP and ALP activities increased by 4.58 and 17.88%, respectively, but drought-stressed soybean plants inoculated with AMF increased by 48.41 and 74.75% **(**Table 5**)**.

**Table 5:** Impacts of drought stress on easily (EE-GRSP) and total extractable glomalin related soil protein (TE-GRSP) (mg/g soil) and activities of acid (ACP) and alkaline phosphatases (ALP) (µmol *p*NP/min) in the shoots of soybean grown under three irrigation regimes

Irrigation regime	AMF	EE-GRSP (mg/g soil)	TE-GRSP (mg/g soil)	ACP (µmol *p*NP/min)	ALP (µmol *p*NP/min)
FI	–	0.314 ± 0.0083a	0.397 ± .0105b	117.595 ± 3.111ab	77.095 ± 2.039b
	+	0.335 ± 0.0088a	0.435 ± 0.0115a	123.304 ± 3.262a	90.887 ± 2.404a
MD	–	0.274 ± 0.0072b	0.324 ± 0.0085 cd	75.137 ± 1.987e	46.595 ± 1.232e
	+	0.313 ± 0.0082a	0.337 ± 0.0089c	111.512 ± 2.950bc	81.429 ± 2.154b
SD	–	0.266 ± 0.0070b	0.301 ± 0.0079d	94.220 ± 2.492d	52.470 ± 1.388d
	+	0.273 ± 0.0072b	0.322 ± 0.0079 cd	105.054 ± 2.779c	68.345 ± 1.808c

Data presented represent the mean of 5 replicates with standard error. Different letters indicate significant differences among treatments using a one-way ANOVA followed by the Duncan’s multiple range test (*p* < 0.05)

FI full irrigation, MD mild drought, and SD severe drought

#### Glomalin content

Table 5 showed that both AMF inoculation and drought stress treatments significantly affected soil GRSP concentrations. Also, AMF inoculation boosted levels of both T-GRSP and EE-GRSP, despite drought reductions under various water irrigation regimes. AMF colonization significantly boosted EE-GRSP concentrations by 14.2% during mild drought stress. While severe drought lowered T-GRSP concentration by 30.8% when compared to the control group. Conversely, the increase in EE-GRSP as well as T-GRSP content is superior in soils of AMF soybean plants growing at control soil moisture levels than in water deficient conditions.

#### PAs biosynthesis genes in soybean genome and expression analysis

To determine the number of PAs biosynthesis genes in soybean, an in-silico screening of soybean genome data base was conducted. The genome survey revealed the presence of one copy of ADC gene on chromosome 6. Two copies of ODC; ODC1 and ODC2 were identified on chr 4 and 6. The search revealed 12 copies coded for spermidine synthase (SpD) that designated as SpD1 and SpD2 and two additional forms of SpD1 like and SpD2 like. There were 3 copies of spermine synthase (SpS) was identified in which one of them was spermine synthase like. Three copies of thermospermine synthase ACAULIS5 genes were identified and only one copy of S-adenosylmethionine decarboxylase (SAMDC) on chr 2 (Table 6).

**Table 6:** Genome basic information about polyamines biosynthesis genes in soybean according to the NCBI GeneBank

Gene	Gene ID		Chromosome #	Coordinates	Transcript ID	Exons #	Transcript length (bp)	Protein ID	Protein length (aa)
Argnine decarboxylase (ADC)	548071		6	NC_038242.2 (579552.-582388)	NM_001251430.2	1	2837	NP_001238359	691
Ornithine decarboxylase (ODC1)	548088		4	NC_016091.4 (1592531.-1594396)	NM_001251700	1	1866	NP_001238629.1	467
ODC2	547682		8	NC_038242.2 (1528720.-1530286)	NM_001249411	1	1590	NP_001236340.1	434
Spermidine synthase1	100812978"		6	NC_038244.2 (39448109.-39456756)	XM_003531964.5	10	1383	XP_003532012.1	335
Spermidine synthase-like	100792085		17	NC_038253.2 (7196342.-7201104, complement)	XM_026126300.2	10	3077	XP_025982085.1	337
Spermidine synthase	100809346		5	NC_038241.2 (3148207.-3151378, complement)	XM_006579500.3	9	1491	XP_006579563.1	341
Spermidine synthase 2	100776863		18	NC_038254.2 (20075098.-20079637, complement)	XM_003551965.5	9	1663	→ XP_003552013.1	335
Spermidine synthase 1-like	100792107		2	NC_016089.4 (3122712.-3125797, complement)	NM_001252932.3	9	1301	NP_001239861.1	340
Spermidine synthase 1-like	100790506		1	NC_016088.4 (3301613.-3304787)	NM_001254596.2	9	1381	NP_001241525.1	338
Spermidine synthase 2	100788406		17	NC_038253.2 (7104777.-7108108)	XM_006600588.4	9	1392	XP_006600651.1	334
Spermidine synthase 2	100785862		6	NC_038242.2 (15850082.-15854817)	XM_003528108.5	9	1491	XP_003528156.1	338
Spermidine synthase 2-like isoform X1	100805622		5	NC_038241.2 (3033674.-3035216)	XM_041015898.1	6	877	XP_040871832.1	174
Spermidine synthase 2-like isoform X2	100805623		5	NC_038241.2 (3033674.-3035216)	XM_041015899.1	6	746	XP_040871833.1	132
Spermidine synthase 1-like	121174789		4	NC_016091.4 (43447211.-43448053)	XM_041014915.1	3	569	XP_040870849.1136	136
Spermidine synthase 2-like	112999929		17	NC_038253.2 (22120693.-22122144, complement)	XM_026126349.1	5	294	XP_025982134.1	97
Spermine synthase	100815244		6	NC_038242.2 (10345946.-10350658, complement)	XM_003526650.5	12	− 1630	-XP_003526698.1	362
					XM_006581571.3		− 1539	-XP_006581634.1	
					XM_014776384.3		− 1510	-XP_014631870.1	
					XM_014776385.3		− 1637	-XP_014631871.1	
Spermine synthase	100816988		4	NC_016091.4 (49406735.-49412008)	NM_001255319.2	13	− 1575	NP_001242248.2	362
					XM_014774361.3		− 1529	XP_014629847.1 (X1)	
					XR_001387735.3		− 1438		
					XR_005890973.1		− 1602		
					XR_005890974.1		− 1487		
Spermine synthase-like	100802076		20	NC_038256.2 (28483497.-28502167)	XM_006606757.1	8	819	XP_006606820.1	272
Thermospermine synthase ACAULIS5	100815510		17	NC_038253.2 (37791142.-37796736, complement)	XM_003550209.5	10	1757	XP_003550257.1	340
Thermospermine synthase ACAULIS5	100784972		14	NC_038250.2 (9856855.-9862080)	XM_003544505.5	10	1859	XP_003544553.1	339
Thermospermine synthase ACAULIS5	100781850		10	NC_038246.2 (47776188.-47779215)	XM_003536489.5	10	1462	XP_003536537.1	334
S-adenosylmethionine decarboxylase (SAMDC)	547981		2	NC_016089.4 (12768377.-12771321, complement)	547981	4	1816	NP_001236931.1	355

**Fig. 6:**
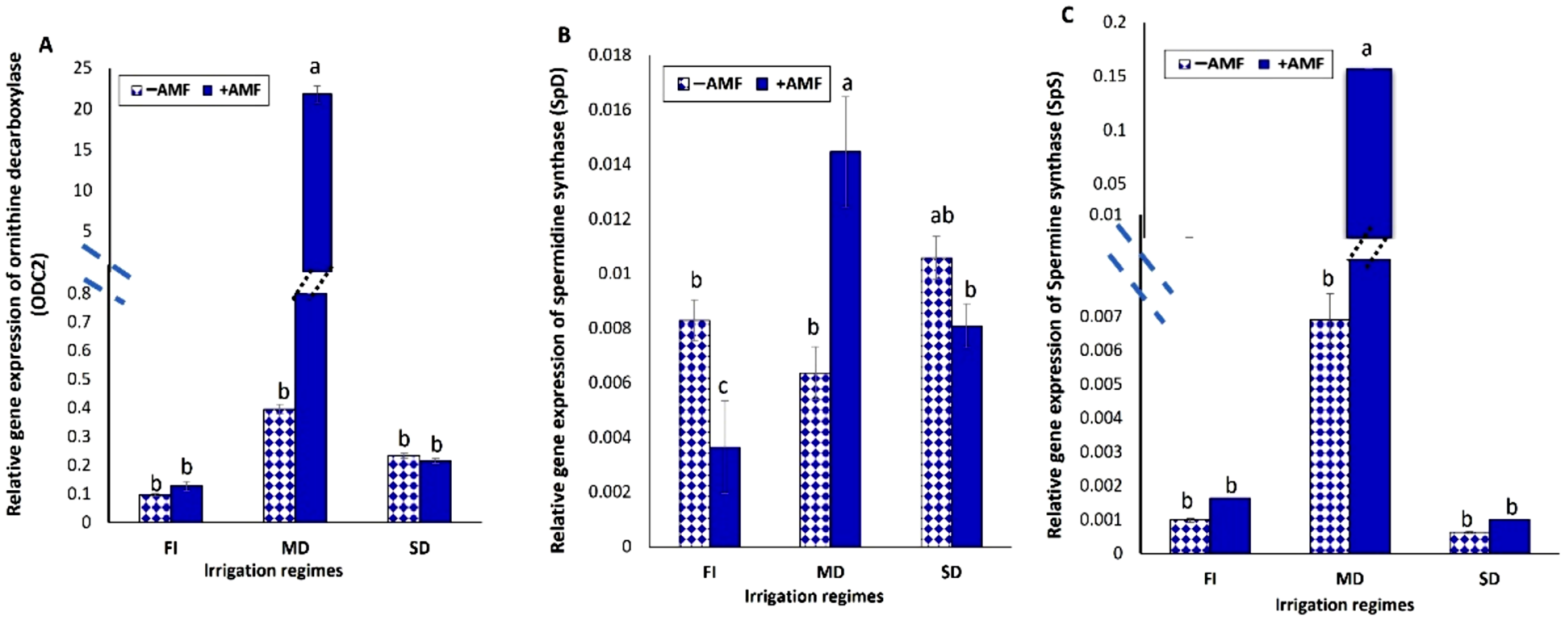
Relative gene expression of PAs biosynthesis genes in response to different treatments. **A** Ornithine decarboxylase, **B** Spermidine synthase (SpD), **C** Spermine synthase (SpS). Data presented represent the mean of 5 replicates with standard error. Different letters indicate significant differences among treatments using a one-way ANOVA followed by the Duncan’s multiple range test (*p* < 0.05)

In soybean shoots of uninoculated plants with AMF, the experience of drought dramatically increased the expression levels of the ornithine decarboxylase gene ODC2, however in shoots of AMF-inoculated plants; there was no discernible upregulation effect in contrast to the control group. The presence of AMF significantly improved the expression of ODC2 in plants exposed to mild drought stress (Fig. 6A). Spermidine synthase (SpD) expression levels were shown to be upregulated during period of severe drought stress, while soybean plants that were inoculated by AMF and exposed to mild drought exhibiting the highest level of expression (Fig. 6B). Spermine synthase (SpS) gene expression levels were significantly boosted in mild drought stressed plant and was even further boosted upon AMF inoculation. Conversely, severe drought stress cause non-significant changes (Fig. 6C).

#### Pearson correlations, hierarchical clustering and principal component analysis (PCA)

**Fig. 7:**
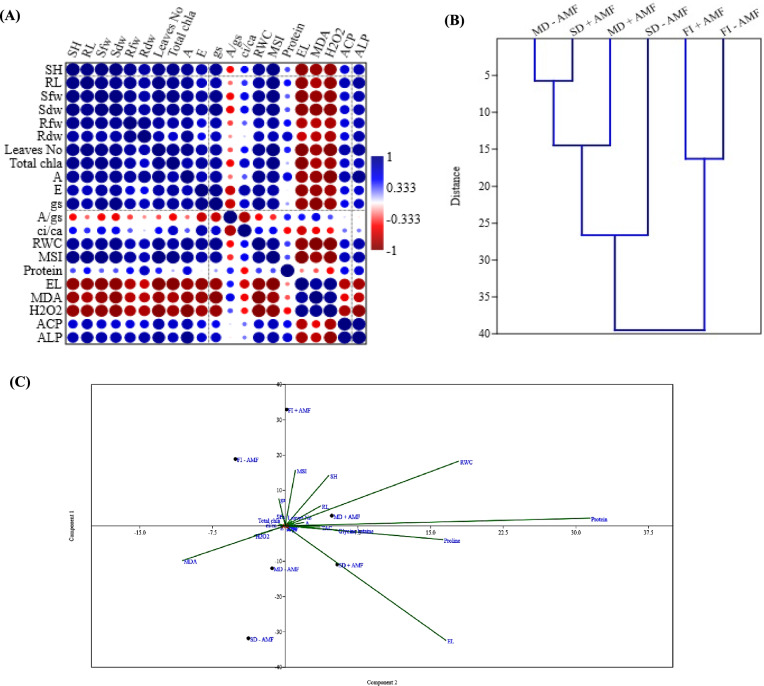
Data inter-relationship analysis **A** Pearson correlation between different parameters, **B** Hierarchical clustering analysis (HCA) between different treatments and **C** Scatter plot of principle component analysis (PCA) analysis between different parameters and treatments (biplot)

The heat map of correlation (Fig. 7A) showed a link between morphological characteristics, stress markers, gas exchange parameters, MSI, RWC, and phosphatase enzymes. There was also a strong positive association between growth metrics (shot height, root length, leaf number, Fwt and Dwt of shoots and roots), total Chl., gas exchange parameters (*A*, *E,* and *gs*), RWC, and MSI. Furthermore, stress markers (EL, MDA, and H_2_O_2_) have a negative correlation with all morphological growth parameters, total Chl., and gas exchange parameters (*A, E,* and *gs*).

The hierarchical clustering analysis (HCA) (Fig. 7B) confirmed the degree of closeness and differences across treatments, revealing grouping between treatments of FI + AMF and FI-AMF, followed by SD-AMF and MD + AMF treatments. Also, there is a difference between the FI + AMF and MD-AMF treatments. This demonstrates the implication of AMF in promoting growth of the plants, performance, and drought tolerance. The PCA-linked scatter plot revealed a clear difference between the treatments in terms of PC1 and PC2 (Fig. 7C). Furthermore, the PCA-associated loading plot revealed that shoot height, RWC, protein, and MSI were more favorably connected with AMF treatments, while stress markers (H_2_O_2_, MDA, and EL) were associated with non-AMF treatments.

## Discussion

To address the drought stress problem which is becoming progressively prevalent owing to global climate change, our current study hypothesizes that inoculating soybean plants with AMF in drought-prone areas can help lessen the detrimental effects on their physiological characteristics and growth since it is crucial for plants to be able to withstand a variety of stresses (Iqbal et al. 2022**; **Li et al. 2022**; **Oliveira et al. 2022). Exposing non-AMF and AMF-inoculated soybean plants to both severe and mild irrigation water regimes elicited in a reduction of all morphological attributes (Fig. 1 and Table 1), and that severe drought produced a substantial decrease in these parameters as compared to mild drought.

The current findings on the harmful effects of drought stress on soybean plants are in line with recent studies by Hashem et al. (2018)**, **Begum et al. (2019), and Li et al. (2022). Furthermore, along the lines of the current findings, Rasouli et al. (2023) found that dryness dramatically reduced morphological features of summer savory plants; however, AMF application greatly improved these growth markers. Plant growth is negatively impacted by drought because it alters the plant's physiological condition during stress, causing ROS buildup (Dossa et al. 2017). Under well-watered and drought-stressed situations, AMF-inoculated maize and *Bombax ceiba* plants increased in height and dry weight significantly compared to the control (Begum et al. 2019; Li et al. 2022). AMF plants performed better in terms of growth and oxidative stress alleviation (Essahibi et al. 2018). Similarly, AMF plays an important function in ecosystems by cycling nutrients (Heflish et al. 2021; Metwally et al. 2022) and enhancing the branching of the plant root system and plant growth (Abd El-Aal et al. 2021). AMF enhances water absorption and nutrient uptake, particularly N, P, and K, via its hyphae (Metwally et al. 2021). This is due to the mycelium's ability to spread and grow outside the rhizosphere, enhancing absorption by raising the root surface and reducing the diffusion distance, and enabling the root area to absorb more nutrients that are immobile and infrequently reach the plant's roots (Jaleel et al. 2007; Abdelhameed and Metwally 2018; Sheikh-Assadi et al. 2023). Even while the hyphae allow water to travel in both directions, most of the water flows from the AMF hyphae to the plant's transpiration route, which enhances the plant's capacity to absorb water from its roots when its environment is dry (Wu et al. 2013).

According to Simard et al. (2012), the AMF inoculum spreads from the infection site into the soil, where it produces secondary mycelium that can bind plants to the mycorrhizal network and ultimately initiate colonization. AMF inoculation in soybean plants increased colonization behavior (Fig. 2 and Table 2), which is according to the findings of Huang et al. (2017) and Rasouli et al. (2023) in trifoliate orange and summer savory plants. Soybean roots underwent a considerable reduction in the rate of AMF colonization during both mild and severe drought stress. This could be linked to the sluggish spread of fungal hyphae caused by a water deficit that occurs after AMF spore germination (Huang et al. 2017). Our findings are comparable with Gong et al. (2014), who found that water stress repressed AMF colonization in foxtail millet roots. This inhibition may be due to the stressed plants' lower carbon supply (Subramanian and Charest 1995). Our results showed that drought stress impacted AMF's capacity to colonize plants, as demonstrated by a decline in the quantity of AMF structural components (spores, mycelia, arbuscules, and vesicles), as previously reported (Sochacki et al. 2013; Hashem et al. 2018). Drought lessens AMF spore germination besides growth, leading to a significant decrease in the synthesis of new endogenous fungal structures (Huang et al. 2017). In contrast to the present findings, Ruiz-Lozano et al. (1995) and Geneva et al. (2023), drought stress had no discernible effect on the pace at which AMF colonized plants.

Also, MD augmented in drought stressed soybean as compared to those under full water irrigation. These findings are congruent with those of Elhindi et al. (2017) and Metwally and Abdelhameed (2018). AMF has a considerable impact on the enhanced progress of drought-stressed soybean plants, which results in an increase in MD values. Therefore, the fact that AMF colonization enabled the plants to endure the detrimental effects of drought-prone stress may serve as evidence of the ecological significance of AMF colonization for soybean plant growth under drought stress.

In contrast to the treatment that is well-watered, the mild and severe droughts had continuously decreasing values for the examined photosynthetic and gas exchange characteristics (Fig. 3 and Table 3**)** which was attributed to reduced leaf area, impaired photosynthetic machinery as a result of membrane leakage, reduced leaf RWC, *gs* and MSI (Wahid and Rasul 2005**)**. In contrast, the addition of AMF inoculum significantly improved all these parameters. Based on the above findings, we may conclude that AMF inoculation considerably reduced the negative impact of drought stress on photosynthetic machinery. Plants under drought stress enhance the closing of stomata, which is necessary to reduce water deficit and directly disrupts the basic metabolism of the plant. This, in turn, reduces CO_2_ intake, inhibiting photosynthesis and limiting growth **(**Oliveira et al. 2019; Müller et al. 2020; Bharath et al. 2021**)**. Furthermore, dryness causes the formation of ROS by interrupting the photosystem, resulting in the destruction of lipids and Chl. (Hu et al. 2020; Cheng et al. 2021**)**.

Moreover, soybean *WUE* rose dramatically with drought and AMF inoculation (Fig. 3D**)**. Nonetheless, their applications resulted in a lower *Ci/Ca* ratio **(**Fig. 3E). These outcomes are comparable with those of Oliveira et al. (2022), who found a significant increase in *WUE* following AMF or drought stress treatments. Furthermore, they asserted that in both well-watered and drought challenged situations, plants infected with AMF exhibited superior photosynthetic rates than non-inoculated plants.

The decreased Chl. content observed in soybean plants under drought stress and the capacity of AMF to stop this depletion corroborate the results of Al-Arjani et al. (2020) in *E. foliata*. Similar findings were made by Oliveira et al. (2022) and Desoky et al. (2023), who showed that osmotic stressors lower *A*, *gs*, *E*, and CO_2_ entrance into plant leaves. By speeding up photosynthesis and lowering the formation of ROS, AMF has the ability to boost the production of photos-assimilates (Hashem et al. 2016). Drought stress lowered Mg absorption, a crucial component of Chl., decreased enzyme activity that regulates Chl. biosynthesis, also increased the chlorophyllase activity, which promotes Chl. breakdown (Zhu et al. 2017; Metwally and Abdelhameed 2018; Al-Arjani et al. 2020**)** whereas AMF-inoculated plants enhanced its uptake, making it available for integration into Chl. and reducing chlorophyllase activity while increasing the expression of Chl. biosynthesis genes, resulting in increased pigment synthesis under both drought-stressed and non-stressed conditions (Hashem et al. 2018; Abdelhameed and Metwally 2019). Increased photosynthetic pigments help to maintain the photosynthesis membrane, absorb P, and encourage the development of AMF plants in conditions of water stress.

RWC and MSI drastically dropped when soybean leaves were subjected to mild or severe drought as opposed to full water irrigation and the reductions were more apparent under extreme drought stress (Fig. 4**)**. However, AMF colonization improved the RWC and MSI parameters. According to Zou et al. (2017) and Oliveira et al. (2022), in the presence of water deficit conditions, the water potential of soybean seedlings and *Poncirus trifoliata* plants inoculated with AMF increased, which is consistent with our findings. The AMF hyphae associated with soybean root boost water intake, making them a possible ally in maintaining plant water status **(**Abdalla and Ahmed 2021). According to Cheng et al. (2021), during water stress, the area and volume of air that exists between soil particles and roots expands; nevertheless, the presence of AMF compensates for this, enabling water transport. As a result, our findings show that AMF inoculation is a vital technique for soybean improvement and aids in the absorption of more water during drought situations.

The levels of oxidative stress markers (MDA, EL and H_2_O_2_) steadily raised with decreased irrigation regime, with the highest values obtained during extreme drought stress, in contrast, AMF application resulted in a significant drop in their levels (Fig. 5). Uncontrolled ROS, such as H_2_O_2_, are crucial signaling molecules that rise during abiotic stress. However, overproduction under stressful conditions can cause severe oxidative damage to cellular membranes by increasing lipid peroxidation, protein and DNA oxidation in plants (Chen et al. 2017; Nasrallah et al. 2020; Ahmed et al. 2021; Metwally and Soliman 2023). Thus, the build-up of ROS and MDA levels in cells may function as widely accepted markers of oxidative damage in plants. Improved enzymatic and non-enzymatic antioxidants lower plasma membrane lipid peroxidation and regulate cellular homeostasis (Heidari et al. 2021). The measurement of MDA and H_2_O_2_ data, which were congruent with the morphological features, revealed that non-AMF inoculation plants accumulated much more of these components than AMF inoculated ones. This shows that inoculation with AMF reduced drought-induced oxidative damage on soybean roots, resulting in an improved response of AMF plants to water deficit conditions.

Further, the levels of osmo-protectants in soybean plant leaves under severe water deficit conditions were significantly greater, with the highest values obtained for AMF-treated plants (Table 4**)**. The pattern of metabolite accumulation demonstrated strong negative correlations with MDA, EL, and H_2_O_2_. In light of this, these osmo-protectants protected soybean plants from oxidative damage and may act as free radical scavengers. Similar findings have been reported in response to AMF application by Begum et al. (2019)**; **Desoky et al. (2021) and Rasouli et al. (2023). Similarly, Abd-Elghany et al. (2021) and Spinoso-Castillo et al. (2023) found that AMF inoculation in plants stressed by the drought produced the highest levels of proline and glycine-betaine in *Ocimum basilicum* and sugarcane. The increased accumulation of osmo-protectants in AMF-treated plants causes a stronger water potential gradient, which overcomes the osmotic imbalance. Stated differently, plants may be protected against the oxidative damage that ROS cause to membranes and proteins by AMF-mediated proline and glycine betaine buildup, leading to increased protein levels (Hashem et al. 2016). Thus, soybean plants treated with AMF were less affected by drought stress by decreasing H_2_O_2_ concentration, MDA, and EL (Sarker and Oba 2020). This supports the idea that proline and glycine-betaine are biochemical signs that donate to soybean drought stress tolerance, explaining the improved growth features observed in AMF infected plants under both normal and drought-stressed circumstances.

Glycine-betaine acts as a compatible osmolyte, increasing antioxidant activity (Hernández-Pérez et al. 2021). It can also protect Rubisco and photosystem II enzyme function during photosynthesis, in addition to maintaining membrane stability and cellular osmotic regulation (Masood et al. 2016; Spinoso-Castillo et al. 2023). Lu et al. (2007) and Wu et al. (2013), some plants create significant proline amounts to improve osmosis and minimize dehydration, which acts as an osmotic buffer, facilitates water absorption, and stabilizes intracellular structures. Furthermore, proline protects the enzymes involved in Chl. production. However, it acts as a carbon and nitrogen storage for plants inoculated with AMF, its elevated concentration may provide the necessary energy for plant growth in the event of drought stress (Hare and Cress 1997; Rasouli et al. 2023). On the contrary, a study by Nader et al. (2024) showed a decrease in proline accumulation due to AMF applications under drought stress. These contradictory results could be caused by variations in plant species, drought severity and duration, and environmental factors (Ye et al. 2023).

As well, water stress induced the greatest increase in TAC, which is proportionate to the severity of drought. This is consistent with previous findings for summer savory (Rasouli et al. 2023) and sugarcane plantlets (Spinoso-Castillo et al. 2023). Furthermore, Talbi et al. (2020) ascertained that the TAC of Saharan plants increased by about 2–3 times as the intensity of drought stress increased.

The colonization profile of mycorrhiza was completed with assessing the ACP and ALP activities, which corresponded to AMF root colonization (Table 5). AMF affects phosphate nutrition via boosting phosphatase enzyme activity, which ensures effective P absorption, assimilation, besides metabolism (Abd_Allah et al. 2015; Al-Arjani et al. 2020; Metwally et al. 2021). ACP and ALP activities were dramatically increased in soybean plants infected with AMF, but were significantly reduced by drought stress. These findings are consistent with Metwally and Abdelhameed (2018) and Abdelhameed and Metwally (2019), who previously observed a decrease in ALP and ACP activity in *Trigonella foenum-graecum* plants exposed to NaCl and Cd stress. The AMF-inoculated plants, which had enhanced phosphatase activity, were more resistant to these stressors. Furthermore, citrus plants injected with AMF exhibited increased soil ACP, ALP, and total phosphatase activity, according to Cheng et al. (2021), regardless of whether they were well-watered or enduring a drought. In contrast to the present findings, Al-Arjani et al. (2020) and Geneva et al. (2023) stated that water deficit conditions cause an enhancement for both ACP and ALP in *E. foliata Boiss* and *Physalis peruviana* L. ALP is generated by bacteria, fungus, and earthworms and functions catalytically above pH 7, Maseko and Dakora (2013) define ACP as plant-derived enzymes. By raising the P availability, augmented phosphatase activity in AMF-inoculated soybean under drought stress contributes to the plants' increased ability to withstand water stress (Stancheva et al. 2008).

Glomalin is a very stable glycoprotein known as glomalin-related soil protein (GRSP), which is only secreted by AMF hyphae and spores and persists in soil (Rillig 2004). It is divided into TE-GRSP as well as EE-GRSP (Driver et al. 2005). In order to reduce water loss within soil aggregates, GRSP covers fungal hyphae and creates a hydrophobic coating on the surface of the aggregate (Nichols 2008). Slowly, GRSP breaks down in the soil. They are essential for the accumulation of soil organic carbon, the enhancement of soil temperature and water, the stabilization of soil aggregates, and the control of plant growth and community development (Li et al. 2022). Our findings showed AMF inoculation boosted levels of both TE-GRSP and EE-GRSP, despite drought reductions under various water irrigation regimes (Table 5). Our findings are consistent with Cheng et al. (2021), who reported that the existence of AMF increased both EE-GRSP as well as TE-GRSP levels while drought stress lowered their contents, implying that soil drought reduces GRSP formation.

While Cheng et al. (2022) observed that AMF inoculation expressively augmented the EE-GRSP and TE-GRSP concentrations regardless of water regime, and that these compounds are released into the soil following the death of AMF hyphae, resulting in an increase in GRSP. Augé et al. (2001) found that water-stable aggregates were more prevalent in AMF soils, and were better organized under drought stress than non-AMF soils. As AMF released GRSP, soil moisture increased, resulting in a relatively higher level of water availability. Wu et al. (2013) found that AMF significantly augmented the dispersion of water-stable aggregates in citrus rhizosphere, which was greatly connected with TE-GRSP concentration. Conversely, nevertheless, the increase in EE-GRSP as well as TE-GRSP content is superior in soils of AMF soybean plants growing at control soil moisture levels than in water deficient conditions, showing absence of water hinders their synthesis and content under the rhizosphere. Drought acclimation of AMF-inoculated soybean plants promoted the formation of extra radical hyphae and soil aggregation, perhaps boosting drought resistance by increasing soil water absorption.

At soybean’ genome, it's interesting to note that a gene cluster representing coordinated regulation and expression is formed by most of the recognized genes implicated in the PAs biosynthesis pathway coexisting on chromosome 6 (Bharadwaj et al. 2021). All of the identified PAs biosynthesis genes except for ADC and ODC are composed of multiple exons (Table 6) suggesting the possibilities of alternative splicing forms (Reddy et al. 2013; Singh and Ahi 2022). The distribution of PAs biosynthesis genes in soybeans has been determined to be limited to 11 chromosomes. The SPD gene family displayed a broad and irregular distribution over several chromosomes, resembling those previously found in the wheat genome (Ebeed 2022).

Plants may be able to survive drought by building up PAs (Tyagi et al. 2022; Li et al. 2024). Ornithine decarboxylase is a key enzyme of putrescine (Put) which is the precursor for PAs biosynthesis in plants. According to gene expression study, ODC is the Put biosynthetic pathway in wheat roots during drought stress (Ebeed 2022). The upregulation of ODC2 gene (Fig. 6A) suggested synthesis of more Put in plants that were stressed by drought and inoculated with AMF. Reduced spermidine and spermine concentrations in roots due to drought stress and AMF drought affected *P. trifoliata*, but a rise in Put and cadaverine linked to an upregulation in PA biosynthesis genes, arginine decarboxylase ADC1 and ADC2 (Zhang et al. 2020), which encouraged AMF invasion in roots even more (Wu et al. 2010). Because of this increased expression, it is proposed that higher concentrations of polyamines will be created, which may help plants further resist the damaging effects of drought stress. Exogenous leaf spraying with Put reduced drought stress in wheat and lettuce by improved water status condition, proline, Chl, amino acid content, and amount of soluble sugar (Gupta et al. 2012; Zhu et al. 2019; Wasaya et al. 2022).

Both Spermidine synthase (SpD) and Spermine synthase (SpS) gene expression results (Fig. 6B and C**)** indicated significant upregulation in mild drought stressed plant that was even further boosted upon AMF inoculation suggested a role of AMF inoculation in drought stress mitigation and that its effect is dose dependent. In tomato plants, Putrescine (Put), spermidine (Spd), and spermine (Spm) concentrations rise collectively for up to 72 h in drought and up to 48 h in salinity treatments. Tomato plants can therefore withstand saline stress and drought for up to two and three days, respectively (Upadhyay et al. 2021**)**. Spraying finger millet plants with 0.2 mM Spd during the early flowering stage prevented the plants from degrading Chl., and they also produced less electrolyte leakage, H_2_O_2_, and caspase-like activity than plants that were not stressed. Additionally, proline accumulation helped to compensate for the water deficit (Satish et al. 2018). Similarly, drought stress response mitigation was observed in different wheat varieties upon exogenous Spd application that was associated with alternative metabolic profiling. The quantitative gene expression profiling revealed up-regulation in ADC, ODC, SpD and SpS synthase genes in Spd treated drought stressed wheat seedlings (Gholizadeh et al. 2022**)** verifying PA's function in minimizing the negative consequences of drought stress. When applied topically to damask rose, Spm or Spd (0.5 mM) increased the plants' relative water and Chl. levels and *gs* in water-stressed plants. In rose plants, proline levels and antioxidant enzyme activity additionally were elevated. Increased resistance to water stress was achieved by the external administration of spermine or spermidine, which also caused modifications in PA metabolism as well as the mechanism that activates antioxidants (Hassan et al. 2018).

## Conclusion

This study provides novel insights into the role of AMF in enhancing drought resilience in soybeans, focusing on multiple stress-responsive mechanisms, highlighting the intricate interplay between morphology, physiology, osmolyte accumulation, antioxidant defense, and polyamine biosynthesis genes, unlike previous studies that primarily examined AMF-mediated drought tolerance through individual traits. As concluded in this study, drought inhibits gas exchange parameters, RWC, and Chl. production in soybean plants while increasing oxidative stress indicators as MDA, EL, and H_2_O_2_ levels. Up-regulation of the host's osmolytes, phosphatases enzymes, mycorrhizal colonization, glomalin contents, antioxidant capacity, and PAs biosynthesis genes served as a defense mechanism that aided in the quick suppression of ROS, thereby avoiding oxidative damage to membranes and proteins, and significantly lessened the negative effects of drought stress on AMF-inoculated plants. These findings have significant implications for sustainable agriculture, particularly in the context of climate change and increasing water scarcity. AMF inoculation presents a natural, eco-friendly alternative to mitigate drought stress, reducing reliance on chemical inputs while improving plant growth. Looking ahead, future research should focus on field-based validations across diverse soil and climatic conditions, explore genotype-specific AMF responses, and investigate potential synergies between AMF and other biostimulants or microbial consortia. Additionally, advances in molecular breeding and gene-editing techniques could help harness the AMF-induced regulatory networks for developing drought-resilient crop varieties. Finally, we recommend using AMF into climate-smart agricultural systems for safeguarding crops, enhancing drought resilience and promoting sustainable soil health and resource conservation.

## Supplementary Information

Additional file 1.

## Data Availability

The relevant datasets supporting the results of this article are included within the article.

## Abbreviations

ACP: Acid phosphatase
ALP: Alkaline phosphatase
AMF: Arbuscular mycorrhizal fungi
*A*: Net photosynthetic rate
*A/gs*: Intrinsic water-use efficiency
*ci/ca*: The ratio of internal to atmospheric CO_2_ concentration
*E*: Transpiration rate
FC: Field capacity
*gs*: Stomatal conductance
MDA: Malondialdehyde
MD: Mycorrhizal dependency
MSI: Membrane stability index
ODC: Ornithine decarboxylase
PAs: Polyamines
Put: Putrescine
RWC: Relative water content
Spd: Spermidine
SPDS: Spermidine synthase
Spm: Spermine
SPMS: Spermine synthase
TAC: Total antioxidant capacity

## Declarations

### Experimental research and field studies on plants

All relevant institutional, national and international guidelines and legislation were compiled or adhered to in the production of this study.
