# Supplementary material for: Role of arbuscular mycorrhizal fungi in drought-resilient soybeans (Glycine max L.): unraveling the morphological, physio-biochemical traits, and expression of polyamine biosynthesis genes
Source: Bot Stud. 2025 Mar 17;66:9. doi: 10.1186/s40529-025-00455-1 (PMC11914442; doi:10.1186/s40529-025-00455-1)
Supplement: Supplementary file 1 — Additional file 1. [file 40529_2025_455_MOESM1_ESM.docx]

**Suppl. Table 1:** Primers list used for quantitative gene expression analysis of some polyamines biosynthesis genes in soybeans leaves using real-time PCR.

| **Gene ID** | **Gene code for** | **Forward primer 5’-3’** | **Reverse primer 5’-3’** | **Product (bp)size** |
| --- | --- | --- | --- | --- |
| GmODC2 | Ornithine decarboxylase, [NM_001249411.2](https://www.ncbi.nlm.nih.gov/entrez/viewer.fcgi?db=nucleotide&id=1261214348) | TCTCATTTACCAATTAAAAATGCCT | AGGCCCATTTGTCCATGAGG | 251 |
| GmSpD | Spermidine synthase, [XM_041018503.1](https://www.ncbi.nlm.nih.gov/entrez/viewer.fcgi?db=nucleotide&id=2027482874) | AAATATTTGCACAAAGAGAGGCT | CGTATGACCCCTCCATCACC | 253 |
| GmSpMS | Spermine synthase, [XM_014774361.3](https://www.ncbi.nlm.nih.gov/entrez/viewer.fcgi?db=nucleotide&id=2027459728) | AAGGCAATCTTTTCCAGGTTTGG | TGAAGCCTTGGCCTTCAGAC | 266 |
| GmACT6 | Gmactin6, NM_001289231) | ATCTTGACTGAGCGTGGTTATTCC | GCTGGTCCTGGCTGTCTCC | 126 |
